# Astragaloside IV Alleviates Intestinal Barrier Dysfunction via the AKT-GSK3β-β-Catenin Pathway in Peritoneal Dialysis

**DOI:** 10.3389/fphar.2022.873150

**Published:** 2022-04-27

**Authors:** Jiaqi He, Mengling Wang, Licai Yang, Hong Xin, Fan Bian, Gengru Jiang, Xuemei Zhang

**Affiliations:** ^1^ Department of Pharmacology, School of Pharmacy and Minhang Hospital, Fudan University, Shanghai, China; ^2^ Department of Nephrology, Xinhua Hospital, Shanghai Jiao Tong University School of Medicine, Shanghai, China

**Keywords:** peritoneal dialysis, intestinal barrier, Astragaloside IV, Akt, GSK-3β, β-catenin

## Abstract

**Background and aims:** Long-term peritoneal dialysis (PD) causes intestinal dysfunction, including constipation, diarrhea, or enteric peritonitis. However, the etiology and pathogenesis of these complications are still unclear and there are no specific drugs available in the clinic. This study aims to determine whether Astragaloside IV (AS IV) has therapeutic value on PD-induced intestinal epithelial barrier dysfunction *in vivo* and *in vitro*.

**Methods:** We established two different long-term PD treatment mice models by intraperitoneally injecting 4.25% dextrose-containing peritoneal dialysis fluid (PDF) in uremia mice and normal mice, which were served as controls. In addition, PDF was applied to T84 cells *in vitro*. The therapeutic effects of AS IV on PD-induced intestinal dysfunction were then examined by histopathological staining, transmission electron microscopy, western blotting, and reverse transcription polymerase chain reaction. The protein levels of protein kinase B (AKT), glycogen synthase kinase 3β (GSK-3β) and β-catenin were examined after administration of AS IV.

**Results:** In the present study, AS IV maintained the intestinal crypt, microvilli and desmosome structures in an orderly arrangement and improved intestinal epithelial permeability with the up-regulation of tight junction proteins *in vivo*. Furthermore, AS IV protected T84 cells from PD-induced damage by improving cell viability, promoting wound healing, and increasing the expression of tight junction proteins. Additionally, AS IV treatment significantly increased the levels of phosphorylation of AKT, inhibited the activity GSK-3β, and ultimately resulted in the nuclear translocation and accumulation of β-catenin.

**Conclusion:** These findings provide novel insight into the AS IV-mediated protection of the intestinal epithelial barrier from damage via the AKT-GSK3β-β-catenin signal axis during peritoneal dialysis.

## Introduction

End-stage renal disease (ESRD) is the final result of the progressive development of chronic kidney disease (CKD), and its incidence has increased in recent years (Yamagata et al., 2015; McCullough et al., 2019; Ruiqi et al., 2021). Peritoneal dialysis (PD) is a renal replacement therapy for patients with ESRD (Mehrotra et al., 2016; Li et al., 2017), allowing patients to undergo dialysis at home, which is cost-effective and highly tolerable. In addition, PD preserves the patient’s residual renal function and ensures better survival compared to hemodialysis (Mehrotra et al., 2016). Unfortunately, long-term exposure to peritoneal dialysis fluid (PDF) with high glucose and high osmotic conditions causes serious complications, including peritonitis, abdominal infection, and intestinal dysfunction (Dong and Guo, 2010; Su et al., 2012; Szeto and Li, 2019), which are the main causes of withdrawal from PD treatment, and can even contribute to deaths. Currently available studies focus primarily on PD-related peritonitis, but little is known about intestinal disorders.

Intestinal symptoms are common in patients with PD, especially disturbed bowel habits, including constipation and diarrhea. Although diarrhea or constipation itself is not a life-threatening complication, they are associated with a decline in the quality of life and may also induce more serious complications. The accompanying colonic mucosal damage, intestinal flora imbalance, and colon inflammation are serious issues that require great attention in patients with continuous PD (Tang et al., 2019; Jiang et al., 2020). Of note, the original intestinal homeostasis is broken, and excess urea is accumulated in ESRD patients. Long-term PDF stimulation would further lead to intestinal barrier injury and increases intestinal permeability, which then increases the possibility of pathogen invasion, a risk factor for peritonitis and abdominal infection (Shu et al., 2009). Studies have also confirmed that most pathogenic organisms are of intestinal origin in patients with continuous PD (Zhang et al., 2022). However, there are no specific drugs available for PD-related intestinal complications. Thus, in-depth studies and new specific drugs for PD-related intestinal complications are crucial.

Astragaloside IV (AS IV) is one of the main active ingredients of the traditional Chinese medicine (TCM) *Astragalus membranaceus*, possessing immunomodulatory, wound healing, and anti-aging properties (Tian et al., 2021). Currently, a prescription containing astragalus has been used to treat chronic constipation in the clinic (He et al., 2020). Emerging evidence has shown that AS IV could alleviate pathological damage to the gastrointestinal mucosa and atrophy in rats (Fan et al., 2016; Jiang et al., 2017; Lee et al., 2017). For chronic kidney disease, AS IV also has the properties of improving renal function (Li et al., 2011; Lu et al., 2020). Although these preceding results imply the biological benefits of AS IV in intestinal disorders and kidney diseases, the efficacy of AS IV in intestinal side effects related to PD remains to be investigated, and the underlying mechanisms need to be explored. Interestingly, most biological effects of AS IV are achieved through activation of protein B (AKT) (Zhang et al., 2011; Du et al., 2018; Li et al., 2019), which plays an important role in the regulation of cell proliferation and apoptosis. Activation of AKT could induce phosphorylation of GSK3β, then inhibits the degradation of β-catenin, which serves as a potential modulator of multiple biological activities (MacDonald et al., 2009; Wang et al., 2017; Banerjee et al., 2019). Based on these findings, we hypothesized that AS IV may regulate the proliferation of intestinal epithelial cells and promote cell junctions to protect intestinal mucosal barrier from long-term PDF exposure.

Herein, we generated *in vivo* and *in vitro* PD-related models of intestinal dysfunction to explore whether AS IV could alleviate intestinal complications, and then to investigate the role of the AKT/GSK3β/β-catenin pathway in the protective activity of AS IV.

## Materials and Methods

### Animals and Animal Models

All animals were obtained from the Shanghai Slaccas Laboratory Animal Co. Ltd. (Shanghai, China). All experimental protocols were approved by the Institutional Animal Care and Use Committee at Fudan University School of Pharmacy and were performed in strict accordance with the guidelines.

Female C57BL/6J mice were maintained at 20–25°C with 12/12-h light/dark cycles and *ad libitum* access to food and water. The experiments began when the mice were 10 weeks old and weighed approximately 20 g. Mice were distributed as follows (n = 6 in each group): control group, PD group, 5/6 Nx group, 5/6 Nx group + PD group, PD + AS IV group, 5/6 Nx + PD + AS IV group. Mice in all 5/6Nx-related groups underwent 5/6 nephrectomy to induce uremia (Tan et al., 2019). The nephrectomy involved the removal of two-thirds of the left kidney and the complete removal of the right kidney, with a 1-week interval between the two resections. After day 0 (before 5/6 nephrectomy) and day 21, serum urea and creatinine levels were measured to confirm the successful modeling of uremia. The mice were then exposed to 2 ml of standard PDF (4.25% g/dL dextrose) daily for a period of 6 weeks in all PD groups (Ferrantelli et al., 2016; Yang et al., 2021). Mice in the PD + AS IV group and the 5/6 Nx + PDF + AS IV group were intragastrically treated with AS IV (10 mg/kg/d, purity ≥98%, Adamas, Shanghai, China) (Jiang et al., 2017; Gao et al., 2020) for 6 weeks.

Male Sprague-Dawley (SD) rats weighing 250 g were used. After 1 week of adaption, 5/6 nephrectomy was performed to induce uremia, then PDF was performed daily for 6 weeks, as described previously. All rats were randomly divided into the following groups (n = 3 in each group): control group, PD group, 5/6 Nx group, 5/6Nx + PD group. Health conditions of animals were checked daily.

### Histological Analysis

After harvesting the animals, the colon was collected for pathological analysis. Colon tissues were fixed in 4% paraformaldehyde overnight and embedded in paraffin. Subsequently, colonic sections were sectioned at a thickness of 4-μm thickness and stained with hematoxylin and eosin (H&E) and periodic acid-Schiff (PAS).

### Transmission Electron Microscopy

For TEM studies, colonic tissues were cut into small blocks and fixed in a cold buffered solution containing 2% glutardialdehyde and 2% paraformaldehyde. Specimens were cut into semi-thin sections (0.5 μm) and ultrathin sections (70–90 nm) from the interested regions and examined using a transmission electron microscope (TEM 109, Zeiss, Jena, Germany).

### Immunofluorescence and Immunohistochemistry

For IF assays, the colon sections were deparaffinized with xylene and rehydrated with consecutive ethanol washes. The samples were incubated with anti-occludin antibody (Abcam, Cambridge, MA, USA) and washed with PBS. T84 cells were incubated with antibody against β-catenin (Proteintech, Rosemount, IL, USA), and nuclei were stained with 40,6-diamidino-2-phenylindole (DAPI). For IHC staining, sections were subjected to antigen retrieval using citric acid buffer and then incubated with primary antibody against β-catenin (Proteintech, Rosemount, IL, USA) at 4°C overnight. After three washes with Tris-buffered saline, the sections were incubated with horseradish peroxidase-conjugated Affinipure goat anti-rabbit IgG. The immunoreactivity was visualized by treatment using the Dako Envision kit HRP (K4006, Dako). The images were captured by a confocal microscope.

### Cell Culture and Treatment

T84 cells were obtained from the Culture Collection of the Chinese Academy of Sciences, Shanghai, China. T84 cells were cultured in DMEM/F-12 (BIOAGRIO, Shanghai, China) medium supplemented with 10% fetal bovine serum (Gibco, Gaithersburg, MD, USA) at 37 °C with 5% humidified CO_2_. After 3-5 passages, T84 cells were seeded into 6-well plates, and experiments were performed when a 50–60% confluence was reached. The cells were exposed to 4.25% dextrose-containing PDF and AS IV (1, 5, and 10 μM) for various time periods. Subsequently, MK-2206 (AKT inhibitor, MedChemExpress, Princeton, NJ, USA) dissolved in DMSO at a concentration of 5 μM was added to the AS IV (5 μM) adminstration group to identify the role of AKT in this setting.

### Cell Viability Assay

The effects of AS IV on T84 cell viability were assessed with the Cell Counting Kit-8 (CCK8, Beyotime Biotechnology, Beijing, China). In detail, 5 ×10^3^ cells/well were seeded in 96-well plates and exposed to various concentrations of AS IV for 24 or 48 h. At the end of the experiment, 10 μl of CCK8 solution was added and co-cultured at 37 C for 2 h, then the absorbance value was calculated at 450 nm.

### Wound Healing Assay

T84 cells were seeded in 12-well plates and scratch wounds were made with a sterile 200 μL pipette tip. The migration of cells into the wound area was observed and photographed at 0 and 48 h using a microscope. The migration distance was measured by ImageJ (Rawak Software Inc. Germany).

### Western Blotting Analysis

Cell and colon tissues were lysed with RIPA buffer containing protease and phosphatase inhibitors and proteins were collected by centrifuging. The proteins were separated by SDS-PAGE and transferred onto a polyvinylidene difluoride (PVDF) membrane (Millipore Corporation, Billerica, MA, USA). The PVDF membranes were then incubated with the corresponding primary antibodies of anti-GAPDH, anti-β-catenin and anti-phosphorylated (p)-GSK3β^Ser9^ (Proteintech, Rosemount, IL, USA); anti-ZO-1, anti-occludin, and anti-p-AKT^S473^ (Abcam, Cambridge, MA, USA); at 4 °C overnight. The membranes were washed 3 times and horseradish peroxidase-conjugated secondary antibodies were added. Finally, specific immunoblots were detected by chemiluminescence. The grey density of the protein bands was normalized to the respective control according to the manufacturer’s instructions.

### RT-PCR

Total RNA from T84 cells was extracted by Trizol reagent (Takara, Japan) according to the manufacturer’s instructions. The cDNA was synthesized using PrimeScript TM RT Master Mix (Perfect Real Time, Takara, Japan), and then each sample was analyzed using a BIO-RAD CFX Connect Real-time PCR system with SYBR Premix Ex Taq TM (Takara, Japan) and the specific primers. Relative quantification of gene expression was performed using the 2^-△△^Cq as fold changes. The designed gene-specific primers are shown in Table 1.

**TABLE 1:** Primer sequences used in the RT-PCR analysis.

Gene		Sequence
GAPDH (human)	Forward	GGA​GCG​AGA​TCC​CTC​CAA​AAT
	Reverse	GGC​TGT​TGT​CAT​ACT​TCT​CAT​GG
Occludin (human)	Forward	AAG​AAG​CCT​ATT​GGA​GCC​ATC​C
	Reverse	TGG​AAC​ACT​GCG​ACA​TAG​CG
ZO-1 (human)	Forward	ACC​AGT​AAG​TCG​TCC​TGA​TCC
	Reverse	TCG​GCC​AAA​TCT​TCT​CAC​TCC
GAPDH (mouse)	Forward	AGG​TCG​GTG​TGA​ACG​GAT​TTG
	Reverse	TGT​AGA​CCA​TGT​AGT​TGA​GGT​CA
Occludin (mouse)	Forward	TGA​AAG​TCC​ACC​TCC​TTA​CAG​A
	Reverse	CCG​GAT​AAA​AAG​AGT​ACG​CTG​G
ZO-1 (mouse)	Forward	GCT​TTA​GCG​AAC​AGA​AGG​AGC
	Reverse	TTC​ATT​TTT​CCG​AGA​CTT​CAC​CA

### Statistical Analysis

All data were analyzed by one-way analysis of variance (ANOVA) or unpaired two-tail Student’s *t-*test with GraphPad Prism (Version 8.0; GraphPad Software, San Diego, CA, USA), and a *p*-value of <0.05 was considered as statistically significant.

## Results

### AS IV Protected the Intestinal Mucosa From Pathological Damage in PD Mice

**FIGURE 1:**
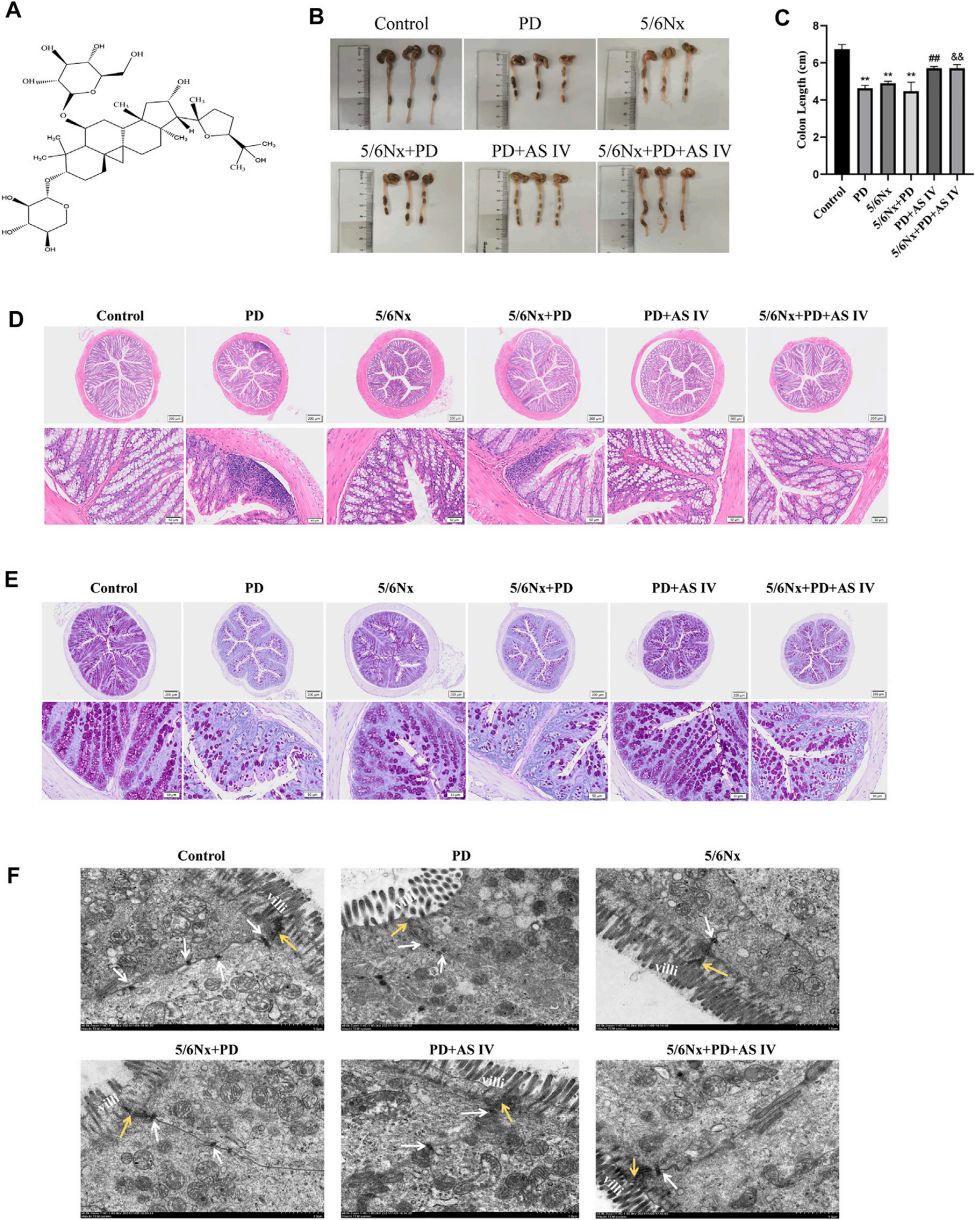
Effects of AS IV on the integrity of the intestinal mucosal epithelium in mice exposed to peritoneal dialysis fluid (PDF) **(A)** Chemical structure of AS IV **(B,C)** Length of the colon **(D)** Representative hematoxylin and eosin (H&E) staining of colonic sections, showing the appearance of the intestinal mucous membranes **(E)** Representative periodic acid-Schiff (PAS) staining of colonic sections, indicating goblet cell morphology and quantity **(F)** Ultrastructure of colonic mucosa observed with a transmission electron microscope. Yellow arrow, tight junction protein; white arrow, desmosomes; villi, microvilli (Scale bar = 1 μM). Data are presented as means ± SD of three independent experiments. *p < 0.05 or **p < 0.01 versus the control group. ^#^ *p* < 0.05 or ^##^ *p* < 0.01 versus the PD group. ^&^ *p* < 0.05 or ^&&^ *p* < 0.01 versus the 5/6Nx + PD group.

We adopted two different mice PD models with uremic mice and normal mice. The prolonged exposure to PDF caused colon length atrophy in the PD group and in the 5/6Nx + PD group (Figures 1B,C). Furthermore, in the PD-related groups, H&E staining showed the infiltration of inflammatory cells, the defects of crypts and the irregular arrangement of the crypts in the colon (Figure 1D) and the PAS staining results revealed the lower number of goblet cells (Figure 1E). These results demonstrated that long-term PDF treatment would cause pathological damage to the colon in normal or uremic mice, which was also confirmed in rat PD models (Supplementary Figure S1). Interestingly, AS IV (Figure 1A) significantly alleviated colon length atrophy, improved crypt morphology, prevented inflammatory infiltration, and increased expression of expression of goblet cells expression (Figures 1D,E). Furthermore, we detected an ultrastructural alteration in the intestinal mucosal epithelium before and after AS IV treatment. TEM of the intestinal epithelium showed intact tight junctions (TJ), orderly microvilli, and assembled desmosome in the control group. After long-term PDF administration, we observed loss of the TJ membrane and desmosome and abnormal appearance of microvilli in the PD group and in the 5/6Nx + PD group, while treatment with AS IV alleviated these distortions (Figure 1F).

### AS IV Improved the Expression of PDF-Damaged Tight Junction Proteins in Mice

**FIGURE 2:**
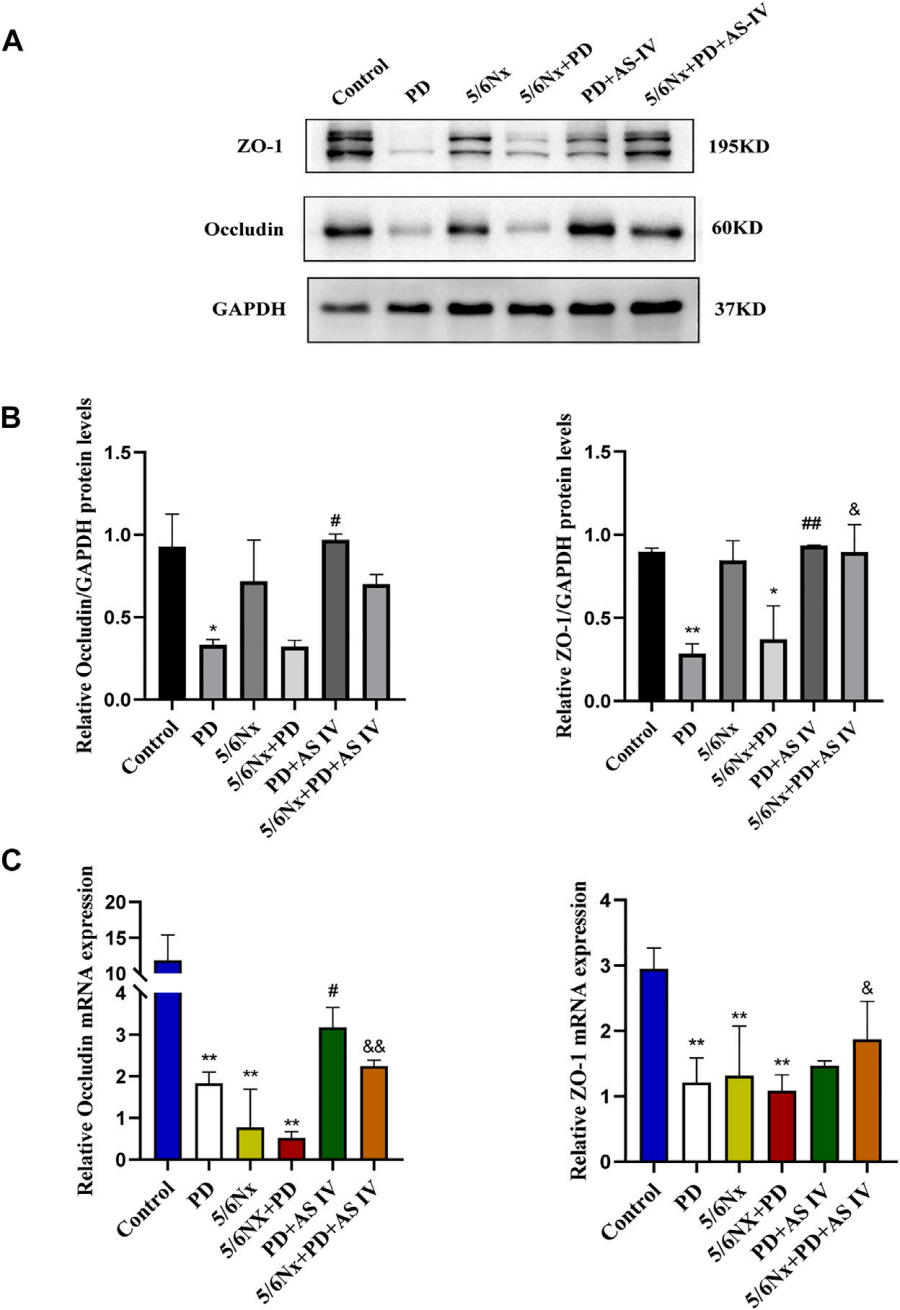
Effects of AS IV on the expression of tight junction proteins in mice exposed to PDF **(A,B)** The protein expression levels of occludin and ZO-1 determined by western blotting analysis **(C)** The mRNA expression levels of occludin and ZO-1 were determined by Real-time PCR. Data are presented as means ± SD of three independent experiments. *p < 0.05 or **p < 0.01 versus the control group. ^#^ *p* < 0.05 or ^##^ *p* < 0.01 versus the PD group. ^&^ *p* < 0.05 or ^&&^ *p* < 0.01 versus the 5/6Nx + PD group.

Intestinal epithelial cells and TJs constitute the intestinal epithelial barrier, which controls the transport of molecules across cells and regulates intestinal permeability (Kumar et al., 2021; Kuo et al., 2021). Here, we performed western blotting and RT-PCR to assess altered levels of TJ proteins, including ZO-1 and occludin, after continuous PDF exposure or AS IV administration. Interestingly, after long-term PDF treatment, the protein expression of ZO-1 and occludin decreased in the PD group and in the 5/6Nx + PD group, but was significantly reversed by AS IV (Figures 2A,B). Furthermore, the RT-PCR results showed a decrease in ZO-1 and occludin expression during PD compared to the control group, but mRNA expression levels were up-regulated after AS IV administration (Figure 2C). Together, these data demonstrated that AS IV could promote the repair of the intestinal epithelial barrier *in vivo*.

### AS IV Promoted Cell Proliferation and Wound Healing in T84 Cells

**FIGURE 3:**
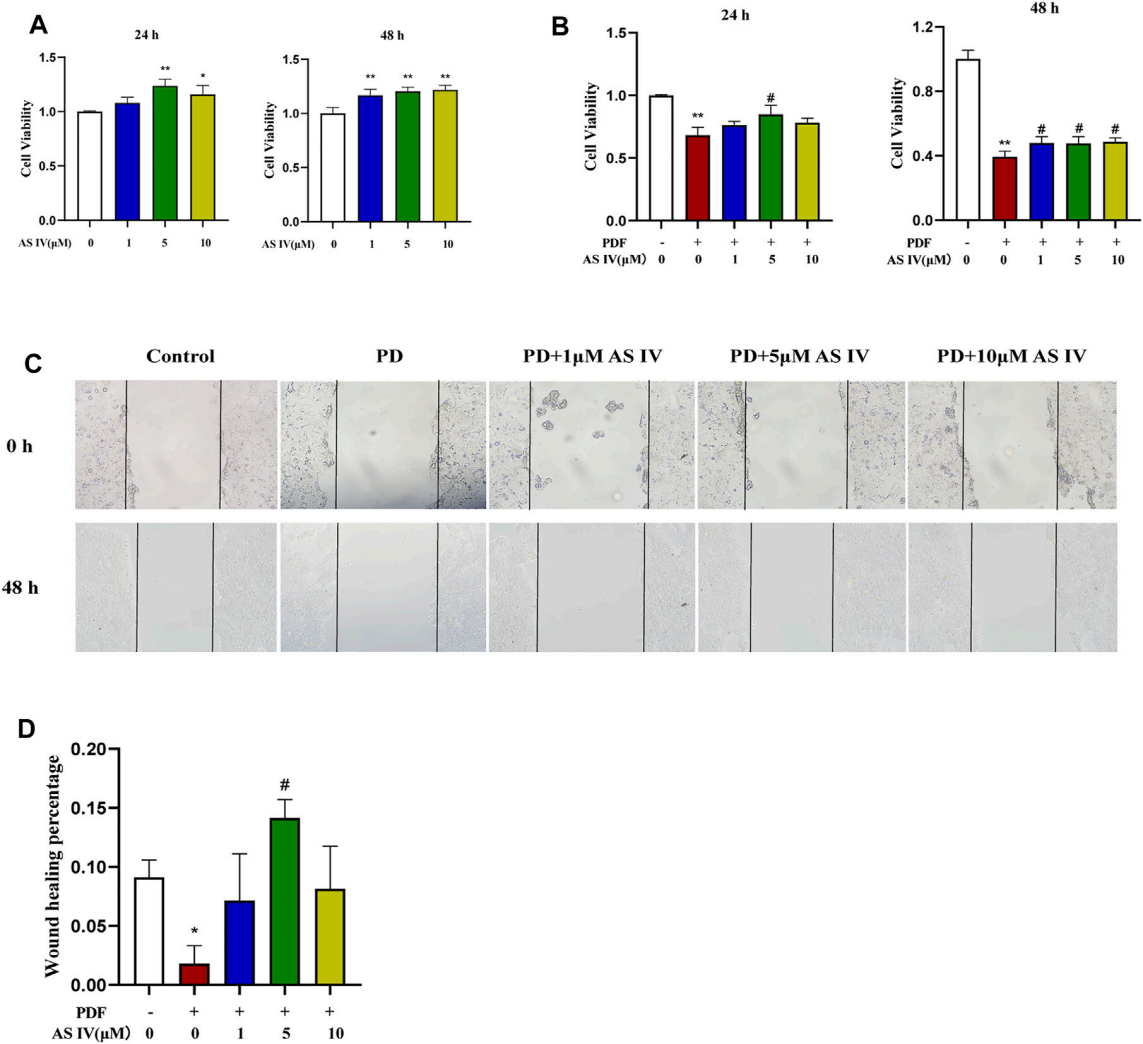
Effects of AS IV on cell viability and scratch wound closure in PDF-treated T84 cells **(A)** T84 cells were treated with 1, 5, and 10 μM AS IV for 24 h and 48 h. Cell proliferation was measured by CCK-8 **(B)** T84 cells were treated with PDF and 1, 5 and 10 μM AS IV for 24 h and 48 h. Cell viability was measured by CCK-8 **(C,D)** Scratch assay of T84 cells incubated with 1, 5 and 10 μM AS IV for 48 h. Data are presented as means ± SD of three independent experiments. *p < 0.05 or **p < 0.01 versus the control group. ^#^ *p* < 0.05 or ^##^ *p* < 0.01 versus the PD group.

We explored the effects of PDF and AS IV on T84 cells *in vitro*. First, we found that AS IV itself could promote T84 cell proliferation and improve cell viability (Figure 3A). As shown in Figure 3B, cell viability decreased dramatically with exposure to PDF, while AS IV (especially at 5 μM) significantly promoted recovery of cell viability in T84 cells. Wound healing is important for the repair of the damaged intestinal epithelial barrier. When PDF was applied to T84 cells for 48 h, cell growth and migration were severely inhibited. Supplementation with AS IV improved the closure of the scratch wound at various concentrations, and maximum efficacy was observed at 5 μM (Figures 3C,D). These results suggested that AS IV promoted intestinal mucosal barrier repair by improving cell activity and promoting wound healing.

### AS IV Increased Tight Junction Expression Levels in PDF-Treated T84 Cells

**FIGURE 4:**
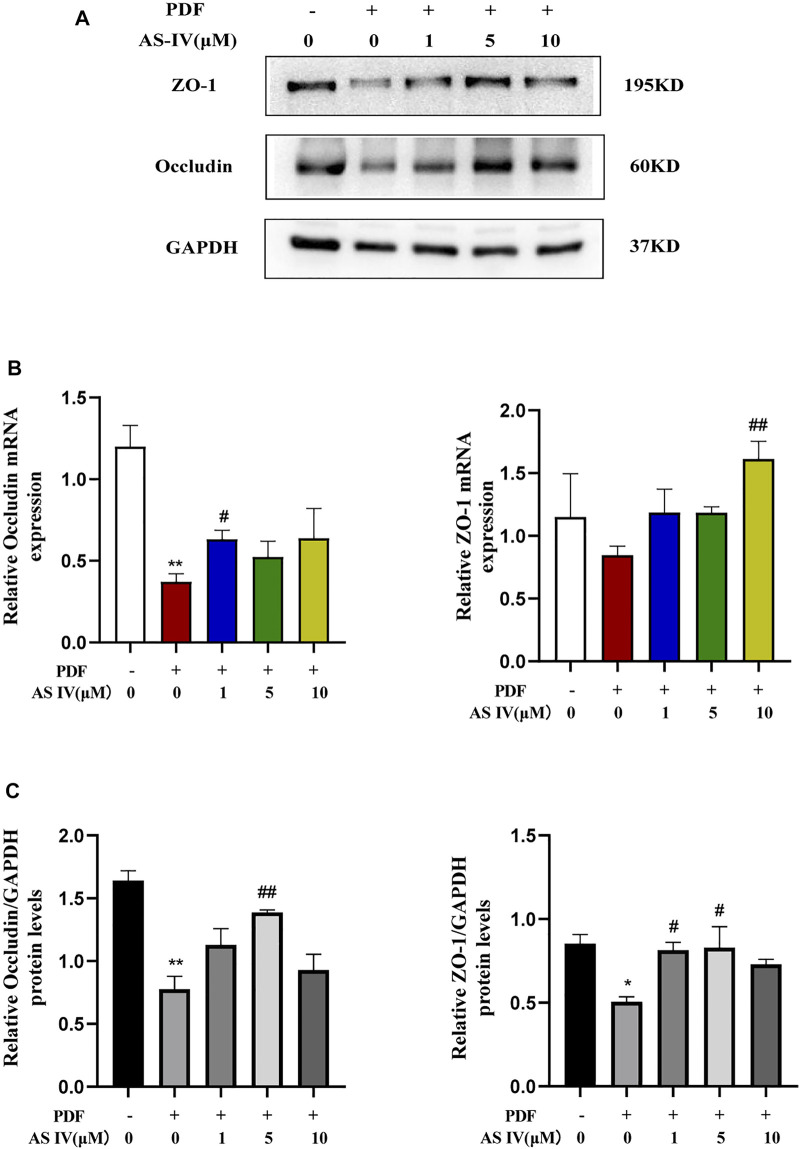
Effects of AS IV on the expression of tight junction proteins in PDF-treated T84 cells **(A,B)** T84 cells were incubated with PDF and 1, 5, and 10 μM AS IV, then the protein expression levels of occludin and ZO-1 were determined by western blotting analysis **(C)** T84 cells were incubated with PDF and 1, 5, and 10 μM AS IV, then the mRNA levels of occludin and ZO-1 were determined by Real-time PCR. Data are presented as means ± SD of three independent experiments. *p < 0.05 or **p < 0.01 versus the control group. ^#^ *p* < 0.05 or ^##^ *p* < 0.01 versus the PD group.

Consistent with the results *in vivo*, the deleterious effect of PDF on TJs was also observed *in vitro*. As shown in Figures 4A,B, after T84 cells were exposed to PDF for 72 h, the expression of TJ proteins, including ZO-1 and occludin, was severely down-regulated and the expression decreased in a dose- and time-dependent manner after PDF exposure (Supplementary Figure S2A, B). Various doses of AS IV were applied for treatment, the expression of the ZO-1 and occludin proteins was significantly up-regulated, which indicated that AS IV could improve intestinal permeability *in vitro* (Figures 4A,B). Furthermore, AS IV also increased the levels of ZO-1 and occludin mRNA *in vitro* (Figure 4C).

### AS IV Exerted Protective Effects Through AKT/GSK3β/β-Catenin Pathway *in vitro*

**FIGURE 5:**
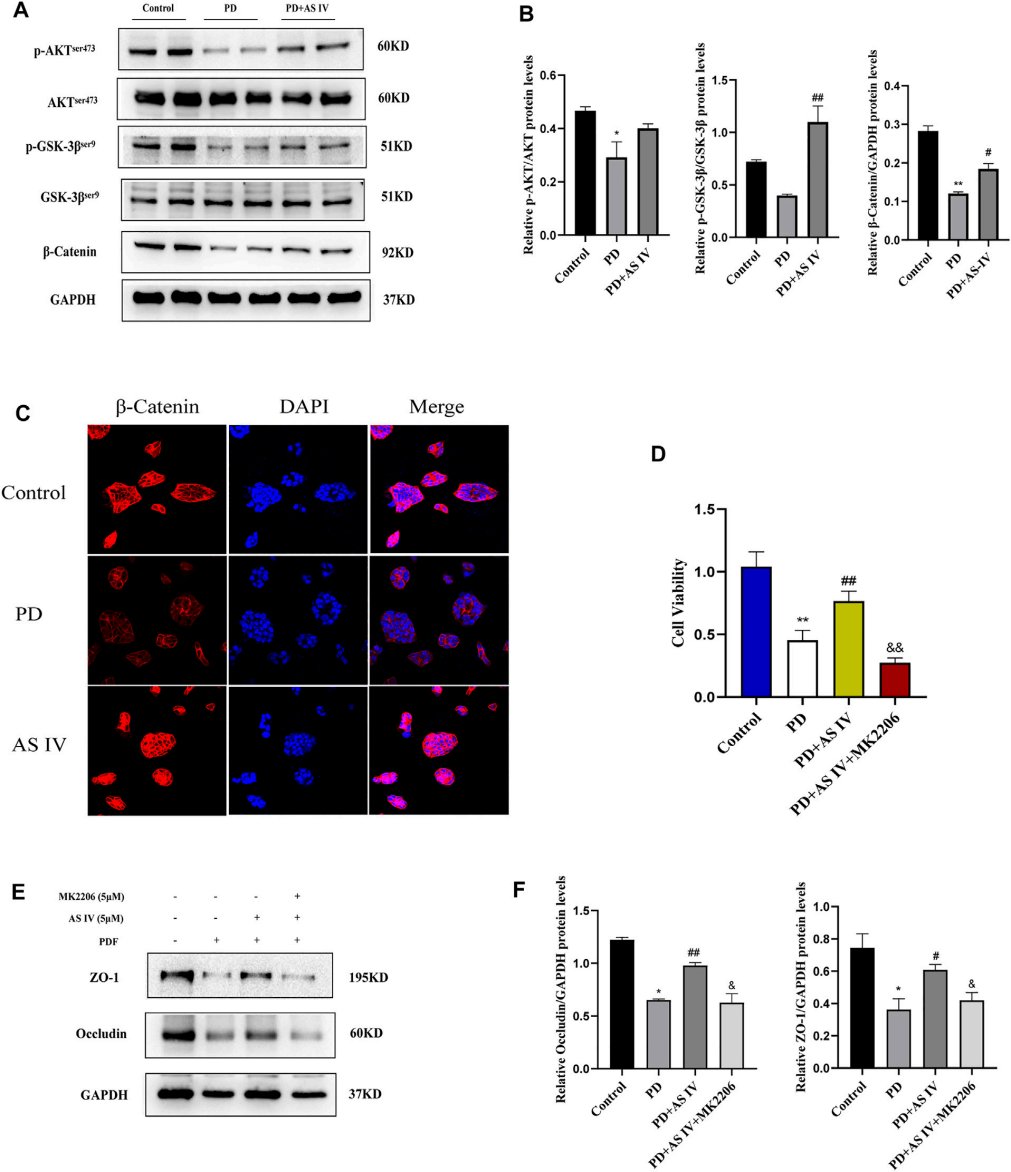
AS IV protected against PD-induced intestinal epithelium damage by activating AKT/GSK3β/β-catenin pathway in T84 cells **(A,B)** T84 cells were incubated with PDF and AS IV (5 μM), then protein expression levels of AKT, p-AKT, GSK-3β, p-GSK3β and β-catenin were evaluated by western blotting **(C)** The distribution and expression of β-catenin were determined by immunofluorescence assay **(D)** T84 cells were incubated with PDF, AS IV (5 μM) and MK2206 (5 μM), then cell viability was calculated by CCK-8 **(E,F)** After the indicated treatments in T84 cells, the protein expression levels of occludin and ZO-1 was determined by western blotting. Data are presented as means ± SD of three independent experiments. *p < 0.05 or **p < 0.01 versus the control group. ^#^ *p* < 0.05 or ^##^ *p* < 0.01 versus the PD group.

To clarify the underlying mechanism whereby AS IV protected T84 cells against PDF-related intestinal epithelial barrier damage, we explored the AKT/GSK3β/β-catenin pathway. As shown in Figure 5A, exposure to PDF reduced the phosphorylation levels of AKT (Ser473) and GSK-3β (Ser9), and concomitantly increased β-catenin degradation. Interestingly, compared to the PDF-treated group, supplementation with AS IV increased AKT phosphorylation levels (Ser473), its activated form, then phosphorylated its downstream GSK-3β (Ser9), the inactivated form. Furthermore, combining the results of western blotting and IF nuclear localization, AS IV subsequently increased the accumulation and nuclear translocation of β-catenin (Figures 5A–C). Therefore, these data demonstrated that AS IV may exert its activity through the AKT/GSK3β/β-catenin pathway. To further verify the underlying pathway, we added 5 μM MK-2206 to the AS IV group. As expected, the addition of MK2206 significantly inhibited the efficacy of AS IV, resulting in a decrease in cell viability (Figure 5D) and downregulated the expression of ZO-1 and occludin (Figures 5E,F). Altogether, these results suggested that AS IV exerted therapeutic effects by improving cell viability and promoting the expression of TJ proteins through the AKT/GSK3β/β-catenin pathway.

### AS IV Activated the AKT/GSK3β/β-Catenin Signaling Axis *in vivo*

**FIGURE 6:**
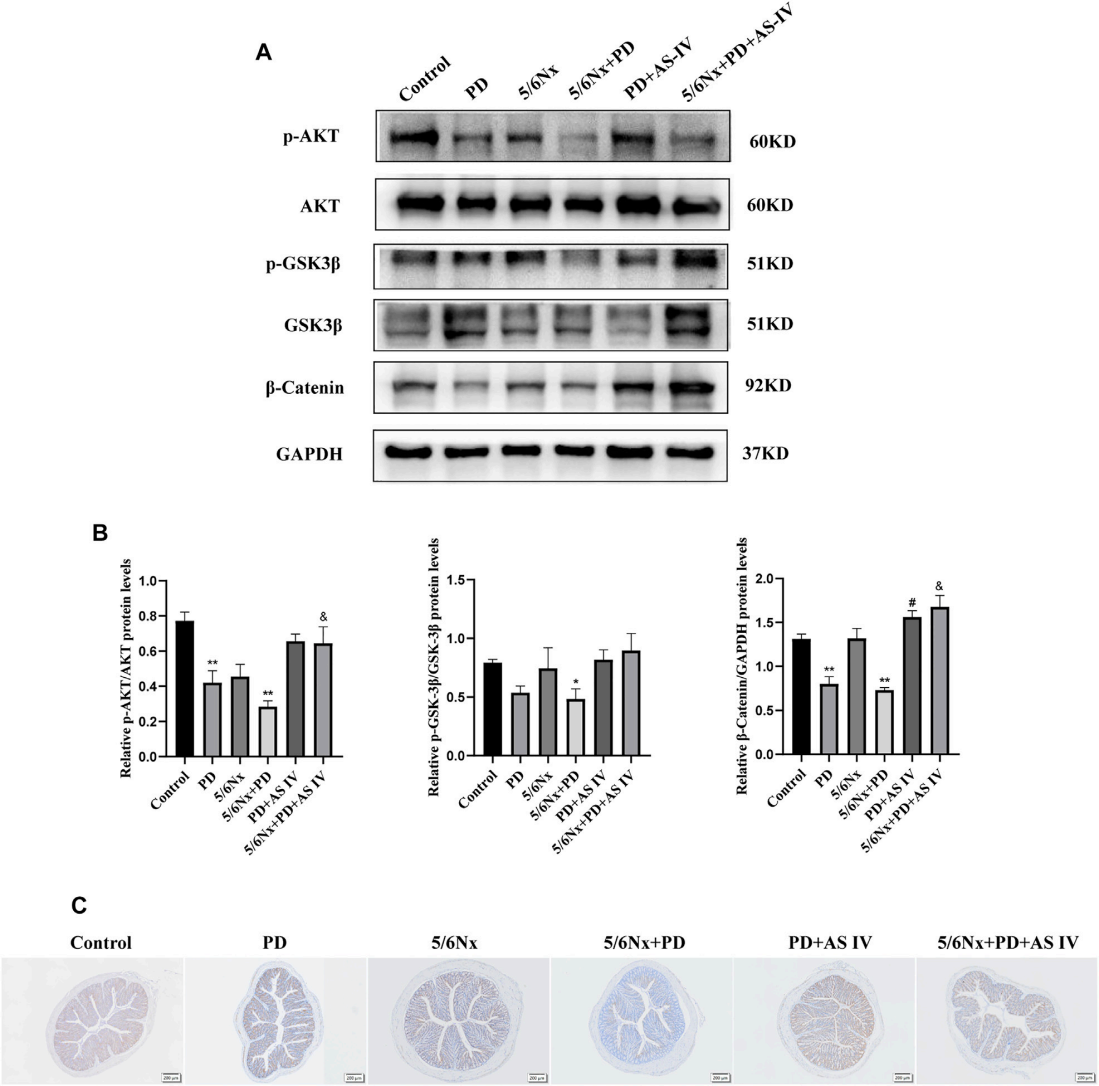
AS IV protected the intestinal mucosa from injury by activating AKT/GSK3β/β-catenin pathway in PD-treated mice **(A,B)** The protein expression levels of AKT, p-AKT, GSK-3β, p-GSK3β, and β-catenin were evaluated by western blotting in colonic lysates **(C)** Representative images of IHC staining for β-catenin in colonic sections. Data are presented as means ± SD of three independent experiments. *p < 0.05 or **p < 0.01 versus the control group. ^#^ *p* < 0.05 or ^##^ *p* < 0.01 versus the PD group. ^&^ *p* < 0.05 or ^&&^ *p* < 0.01 versus the 5/6Nx + PD group.

To validate the underlying mechanism of AS IV, we explored the AKT/GSK-3β/β-catenin pathway *in vivo*. In line with the above findings, western blotting and IHC analyses showed decreased levels of p-AKT (Ser473) and GSK-3β (Ser 9), which was accompanied by a lower expression of β-catenin in the PD group and the 5/6Nx + PD group. However, for the PD + AS IV group and 5/6Nx + PD + AS IV group, we found that administration of AS IV could activate the biological activity of AKT (Ser473), and then inhibit the activity of GSK-3β (Ser 9), ultimately leading to the increased accumulation of β-catenin (Figures 6A–C). These data further confirmed that AS IV promoted the repair of the intestinal mucosal epithelial barrier repair via the AKT/GSK3β/β-catenin signaling axis.

## Discussion

In the clinical setting, approximately 50% of patients with continuous ambulatory PD experience gastrointestinal complications (Kosmadakis et al., 2018), which bring inconvenience to patients, reduce the quality of life, and may increase the risk of enteric peritonitis. Although the incidence of these disorders is high, there are few scientific studies on PD-related intestinal symptoms and no specific drugs for treatment.

AS IV has a wide range of pharmacological effects and has been applied in various disorders, such as colitis, diabetic nephropathy, and CKD (Li et al., 2011; Fan et al., 2016; Jiang et al., 2017; He et al., 2020). In the present study, considering the possibility that AS IV protect intestine from several forms of dysfunction, we innovatively applied AS IV to ameliorate the PD-related intestinal disorders.

Here, we used the 5/6Nx + PD animal model to mimic the clinical situation of patients with continuous ambulatory PD (Van Biesen et al., 2006; Gonzalez-Mateo et al., 2008) and observed the intestinal status *in vivo*. To further investigate the adverse effects of PDF on the intestinal tract, we also established a group of normal mice that were treated with PDF alone for a prolonged period, to exclude the influence of uremia. Interestingly, both normal and 5/6Nx animals experienced severe intestinal epithelial dysfunction after long-term exposure to PDF. Our findings showed that AS IV could improve the morphology of intestinal tissue and protect cellular structures such as desmosomes, and microvilli. The intestinal epithelium is a single layer of columnar epithelial cells that separate the intestinal lumen from the underlying lamina propria (Chen et al., 2019). It is a natural barrier to prevent or inhibit the systemic translocation of pathogens. AS IV could improve intestinal epithelial permeability with the up-regulation of tight junction proteins *in vivo*. Regarding intestinal epithelial cells, AS IV could significantly improve cell viability, promote wound healing, and expression of TJ proteins. This evidence strongly suggests that AS IV can alleviate PD-related intestinal dysfunction. Furthermore, unlike previous studies that only explored the therapeutic effects of the drug in mice treated with PDF alone (Zhang et al., 2021), we also explored the therapeutic effects of the drug on PDF-treated uremic mice, which is more in line with the clinical situation. These results provide scientific evidence and reference for the treatment of intestinal disorders related to PD in the clinical setting.

β-catenin is a well-known component of adherent junctions (AJ), which function as master regulators of the structure of TJ, and loss of β-catenin would lead to disruption of TJ integrity and impair barrier function (Banskota et al., 2018; Pradhan-Sundd et al., 2018). In addition, when β-catenin enters the nucleus, it regulates cell viability and the expression of epithelial junction molecules via the Wnt/β-catenin signaling pathway (Zhang et al., 2017). GSK-3β is a negative regulator of β-catenin, which induces β-catenin degradation. In the present study, we found that AS IV could activate the AKT/GSK3β signaling, then increased the accumulation of β-catenin in the nucleus, which ultimately affected the biological activity of intestinal epithelial cells. To further support this finding, we introduced MK2206 into cultures to block AKT/GSK3β/β-catenin pathway, and found that MK2206 could prevent the proliferation, migration, and interaction of T84 cells, and ultimately attenuated the therapeutic effects of AS IV.

To our knowledge, this study is the first to propose and elucidate the therapeutic role of AS IV in PD-induced intestinal epithelial dysfunction. This protective activity is at least in part, attributed to the AS IV-mediated activation of AKT, followed by inhibition of GSK3β, which ultimately leads to facilitating β-catenin nucleus translocation, regulating cell proliferation, and the expression of TJ proteins. Given the excellent efficacy of AS IV, we speculate that AS IV may be developed as a potential candidate drug for PD-related intestinal symptoms in the clinical setting.

## Data Availability

The original contributions presented in the study are included in the article/Supplementary Material, further inquiries can be directed to the corresponding authors.

## Abbreviations

PD, Peritoneal dialysis; PDF, Peritoneal dialysis fluid; AS IV, Astragaloside IV; ESRD, End-stage renal disease; CKD, Chronic kidney disease; AKT, Protein B; GSK-3β, Glycogen synthase kinase-3β; TJ, Tight junction; 5/6Nx, 5/6 Nephrectomy; TCM, traditional Chinese medicine.
