# Supplementary figures and images for: Astragaloside IV Alleviates Intestinal Barrier Dysfunction via the AKT-GSK3β-β-Catenin Pathway in Peritoneal Dialysis
Source: Front Pharmacol. 2022 Apr 27;13:873150. doi: 10.3389/fphar.2022.873150 (PMC9091173; doi:10.3389/fphar.2022.873150)

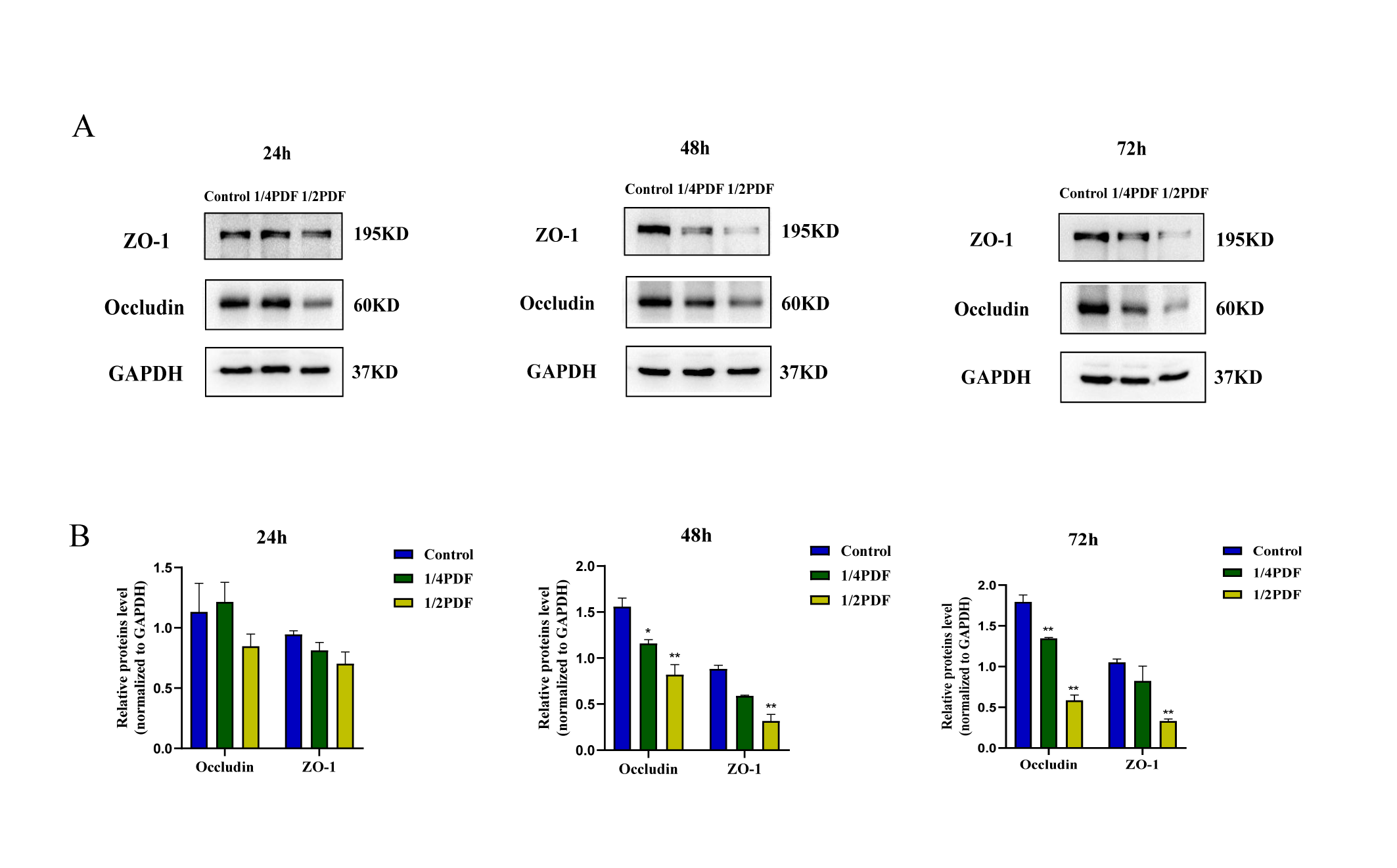

Supplement: Supplementary file 1 [file Image2.TIF]

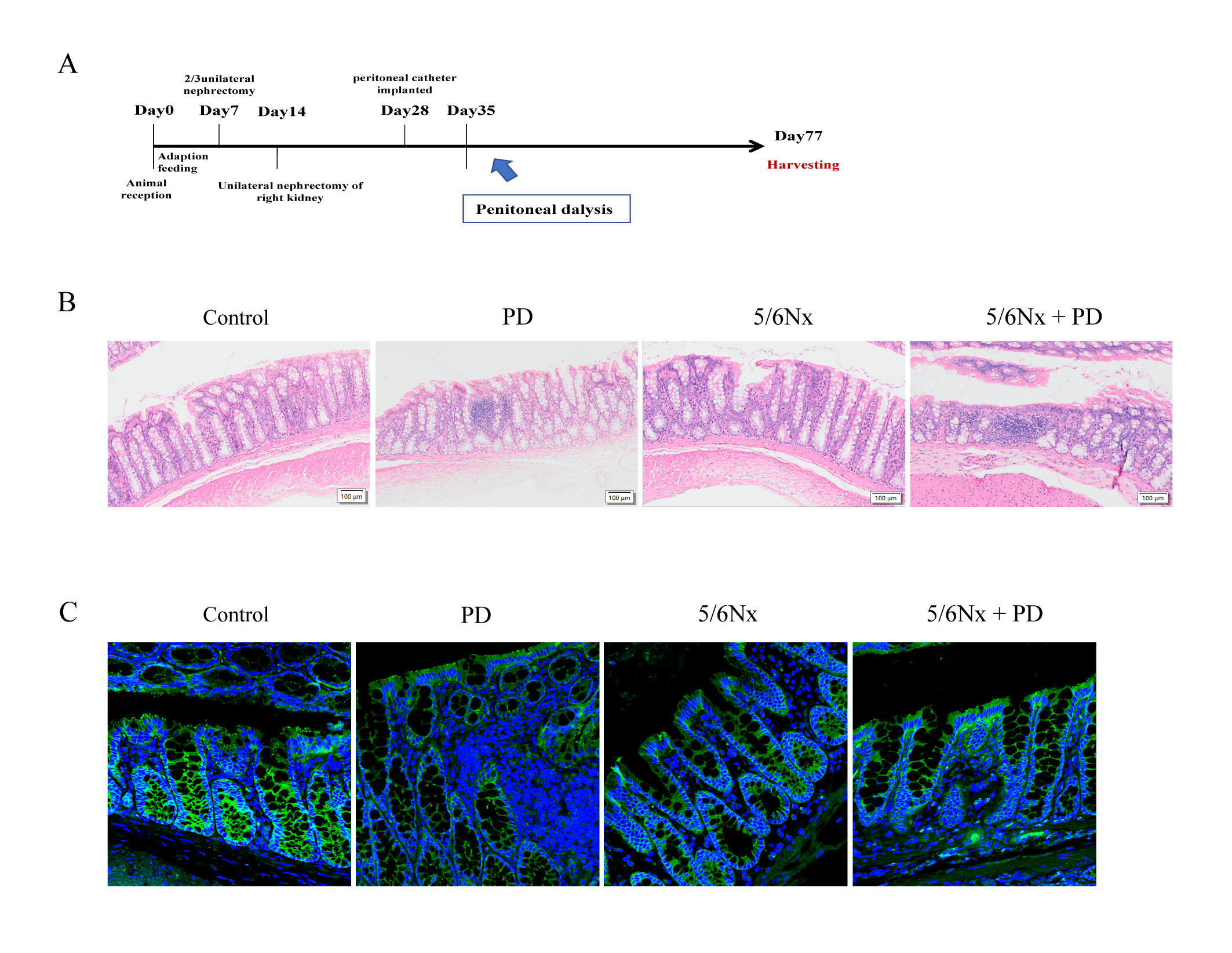

Supplement: Supplementary file 2 [file Image1.TIF]
