# Supplementary material for: Astragaloside IV Alleviates Intestinal Barrier Dysfunction via the AKT-GSK3β-β-Catenin Pathway in Peritoneal Dialysis
Source: Front Pharmacol. 2022 Apr 27;13:873150. doi: 10.3389/fphar.2022.873150 (PMC9091173; doi:10.3389/fphar.2022.873150)
Supplement: Supplementary file 3 [file DataSheet1.PDF]

Supplementary Material

1 Supplementary Figures

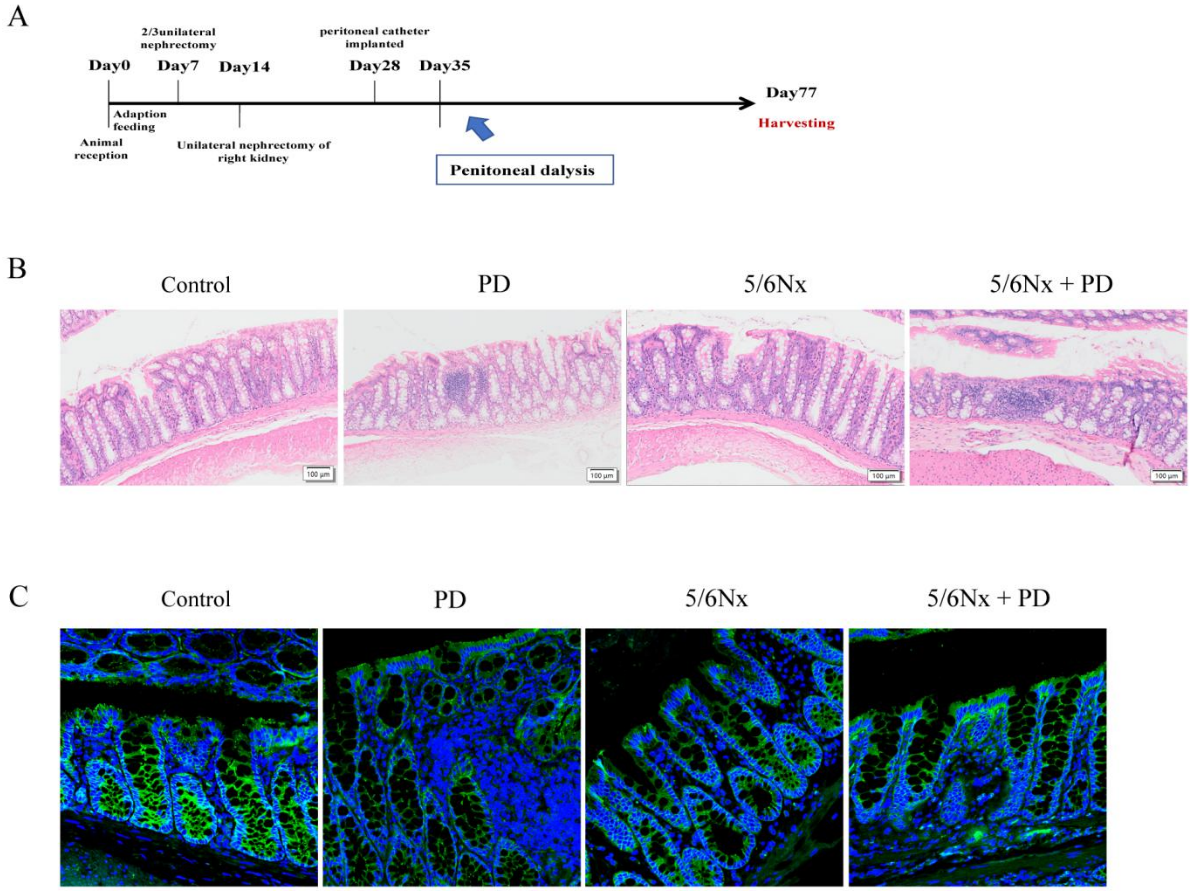

Supplementary Figure 1. Long-term PDF exposure caused intestinal mucosa injury in rats. (A) The design of PD-treated rat models. (B) Representative images of H&E staining of colonic sections in different PD-treated rat models. (C) The expression and distribution of occludin in colon sections were analyzed by immunofluorescence.

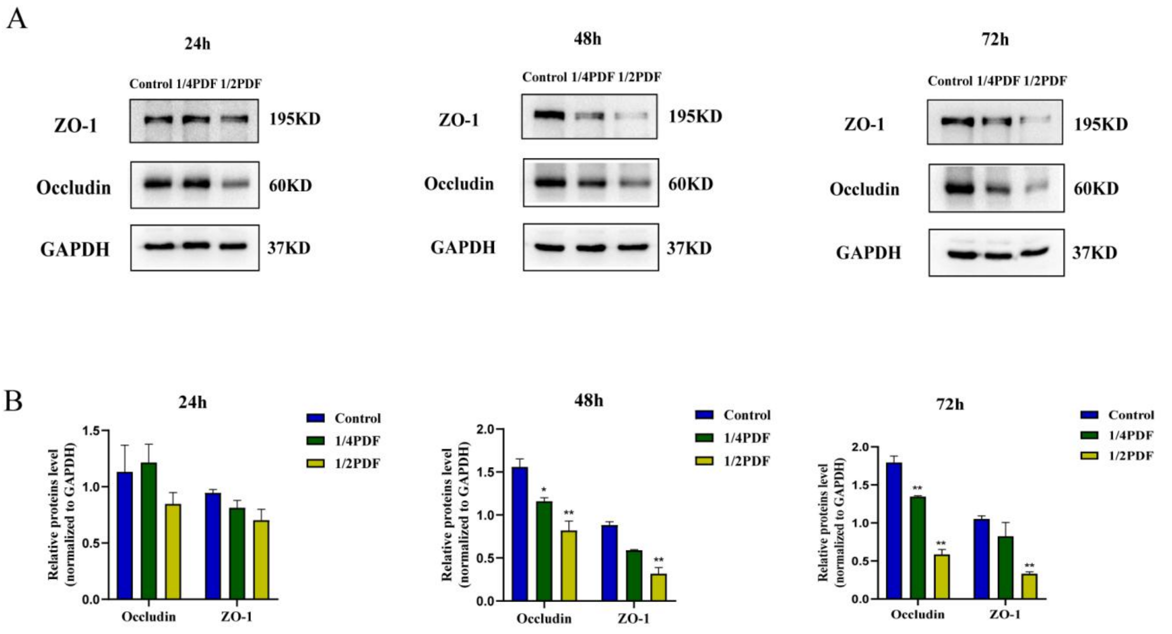

Supplementary Figure 2. PDF treatment decreased the expression of tight junction proteins at different doses and different periods in T84 cells. (A) T84 cells were incubated with PDF and culture medium (1:1 and 1:3) for 24 h, 48 h and 72 h, then the protein expression of occludin and ZO-1 observed by western blotting. (B) The bar chart represent relative protein levels of occludin and ZO-1 compared to the of GAPDH levels. Data are presented as means ± SD of three independent experiments. *P < 0.05 or **P < 0.01 versus the control group.
