# Supplementary material for: Astragaloside IV Alleviates Intestinal Barrier Dysfunction via the AKT-GSK3β-β-Catenin Pathway in Peritoneal Dialysis
Source: Front Pharmacol. 2022 Apr 27;13:873150. doi: 10.3389/fphar.2022.873150 (PMC9091173; doi:10.3389/fphar.2022.873150)
Supplement: Supplementary file 4 [file DataSheet2.docx]

**Raw Data**

Figure 1:

https://www.jianguoyun.com/p/DUwjIcYQkt-hChi3l60E

Figure 2:

https://www.jianguoyun.com/p/DUmlBAMQkt-hChjTl60E

Figure 3:

https://www.jianguoyun.com/p/DcPQZoAQkt-hChjel60E

Figure 4:

https://www.jianguoyun.com/p/DWhP1O0Qkt-hChjll60E

Figure 5:

https://www.jianguoyun.com/p/DdjH4yQQkt-hChjml60E

Figure 6:

https://www.jianguoyun.com/p/DUWDhDUQkt-hChjol60E

Supplementary Figure 1:

https://www.jianguoyun.com/p/DYBOimQQkt-hChjpl60E

Supplementary Figure 2:

https://www.jianguoyun.com/p/DV4GA2cQkt-hChjsl60E
